# Dynamics of Perceived Positive Changes and Indicators of Well-Being Within Different Phases of the COVID-19 Pandemic

**DOI:** 10.3389/fpsyt.2021.685975

**Published:** 2021-06-15

**Authors:** Arndt Büssing, Daniela Rodrigues Recchia, Thomas Dienberg, Janusz Surzykiewicz, Klaus Baumann

**Affiliations:** ^1^Professorship Quality of Life, Spirituality and Coping, Faculty of Health, Witten/Herdecke University, Herdecke, Germany; ^2^IUNCTUS—Competence Center for Christian Spirituality, Philosophical-Theological Academy, Münster, Germany; ^3^Chair of Research Methods and Statistics in Psychology, Faculty of Health, Witten/Herdecke University, Witten, Germany; ^4^Chair of Social Pedagogy, Catholic University Eichstätt-Ingolstadt, Eichstätt, Germany; ^5^Caritas Science and Christian Social Work, Faculty of Theology, Albert-Ludwig-University, Freiburg im Breisgau, Germany

**Keywords:** coping with pandemic stress, perceived changes, well-being, spirituality, COVID-19 pandemic

## Abstract

**Background:** During the COVID-19 pandemic, people reported about fears, depressive states, and phases of loneliness. However, there have also been positively experienced changes in terms of awareness of nature, reflection of life, more intensive relationships, meaningful digital media usage to connect with others, and interest in spirituality. We were interested in the dynamics of these indicators directly after the first lockdown, the summer months and during the second wave of the pandemic with its second lockdown, and how they relate to the perceived restrictions, fears, and worries.

**Method:** Survey with standardized questionnaires, i.e., Perceived Changes Questionnaire, WHO-Five Well-being Index, Brief Multidimensional Life Satisfaction Scale, Awe/Gratitude scale. Participants were categorized as cohort 1 (June 2020; *n* = 1,333), cohort 2 (July to September 2020, *n* = 823), and cohort three (October 2020 to January 2021, *n* = 625).

**Results:** Participants perceived changes in specific attitudes and behaviors, which have impacted their well-being and life satisfaction. Compared to their experiences directly after the first wave of the pandemic (cohort 1), well-being (Hedge's g = 0.83) and life satisfaction (g = 0.63) decreased during the second wave (cohort 3) and participants' stressors increased (g = −0.94). At the same time, positive perceptions such as Nature/Silence/Contemplation (g = 0.67), Spirituality (g = 0.62), Relationships (g = 0.55), and Digital media usage declined (g = 0.31), but not Reflections on life (g = −0.03). In cohort 3, the proportion of persons relying on their faith as a strong hold was declining also in nominally religious persons. Awe/Gratitude was among the best predictors of perceived positive changes, indicating a resource which is nevertheless declining during the second wave of the pandemic (g = 0.60).

**Conclusions:** Several perceptions, attitudes, and behaviors have changed, particularly during the second wave of the pandemic, which had a strong influence on psychological health. Although Awe/Gratitude was confirmed as the best predictor of perceived positive changes, this resource may not buffer against the negative outcomes of the pandemic but helps to recognize the still positive aspects in life. There is a need for new and not yet defined public health communities that could focus on persons which are affected in their physical, mental, social, and spiritual health and well-being due to the pandemic.

## Introduction

During the COVID-19 pandemic, many people have experienced difficult times of social isolation and anxieties about getting infected and suffering from complicated courses of disease (1–4), while others did not share these experiences and ignored social restrictions (5). Although the strategies to cope with the pandemic are in fact heterogeneous (6), one can state that within the general population increased levels of pandemic related stress, anxiety, and depression have been prevalent (7–9). In several cases, persons' mental health deteriorated during the pandemic (10), and others had indicators of “defeat stress” (11). Generally, persons at risk were in fear of the COVID-19 infection and had lower quality of life, e.g., tumor patients (1–4) or pregnant women (12). In adolescents and young adults, it was found that restricted relationships resulted in an increase of negative affect, while loneliness impaired their mental health (9, 13). In Italy, adolescents had a “low perception of risk of COVID-19” during the first wave of the pandemic, while they nevertheless consented that the recommended restrictions may protect others (14). Another important finding of this study was that female students and adolescents living in more severely affected regions “showed more significant psychological negative feelings about the quarantine experience” (14).

Apart from the obvious negative outcomes of the pandemic, there are also reports of positively experienced changes in attitudes and behaviors, particularly in terms of (1) Nature/Silence/Contemplation, (2) Spirituality, (3) Relationships, (4) Reflection on life, and (5) Digital media use (4). These changes were more intensely observed in the elderly, persons with higher well-being, and people who were able to rely on their faith as a resource to cope (4). The best predictor of most of these changes has been the ability to stop in wondering awe in certain situations (these are often related to nature) with subsequent feelings of gratitude (4). These perceived changes can be related to the concept of posttraumatic growth. This implies that in difficult life situations people may perceive differently and change their attitudes and behaviors, i.e., in terms of positive affect, personal strength, appreciation of life, changed priorities, more intimate relationships, prosocial behaviors, and spiritual development (15–17). This change is not simply a buffer against harm (in terms of resisting these affections), but a resilient process of change and of finding meaning in trauma and development (in terms of reappraisal coping). In a similar way, adaptation processes related to the COVID-19 pandemic may result in processes of inner change, as found in the above mentioned perceptions directly after the first lockdown in 2020 (3, 4).

During the summer months of 2020, following the COVID-19 outbreak and first lockdown restrictions, the number of infected and dying persons was decreasing and several restrictions were stopped step by step. In consequence, social distancing and wearing protection masks were practiced less often. Perceived stress declined in this period, and more emotional stability was observed (18). A small though loud fraction of the population protested publicly against the necessity of the official protection measures (19–21), and an optimism bias related to less engagement in behavior changes arose (22). This could be seen as an attempt to protect autonomy in an uncontrollable situation—and accepting the risk to infect others. Thus, the social situation is complex and often contradictory.

During October 2020, however, the number of infected persons was increasing quickly again, and a second lockdown followed in Germany in December 2020 to control the strongly growing numbers of hospitalized patients and persons dying from the COVID-19 infection. Actually, during September and October 2020, it became clear that a second wave of the COVID-19 pandemic started which would be even stronger than the first one. How were people affected by this dramatic development of the pandemic, and how did they cope or change their behaviors—how did they react mentally, emotionally, spiritually? How would these changes compare with the ones perceived in the first wave? To answer this, we analyze the dynamics and interactions of fears and worries, well-being and stressor, and perceived changes in attitudes and behaviors due to the pandemic. Participants of the ongoing survey were categorized due to their survey entry in June 2020 (cohort 1, directly after the first lockdown), during the summer months July to September 2020 (cohort 2), and October 2020 to January 2021 (cohort 3, including the lockdown months December 2020 and January 2021).

## Materials and Methods

### Recruitment of Participants

Participants were recruited via snowball sampling in different networks in Germany, i.e., university students and staff, research collaborators, religious orders and church communities, Rotary Club members, Facebook sites, private websites of public persons, etc., starting in June 2020. In addition, all were invited to spread the information about this survey in their personal networks, too.

Participants were assured confidentiality, were informed about the purpose of the study, and were provided data protection information at the starting page of the online survey. By filling in the anonymous questionnaire, interested persons consented to participate. Neither identifying personal details nor IP addresses were recorded to guarantee anonymity.

### Measures

#### Perception of Changes

To assess which changes of attitudes, perceptions, and behaviors due to the Corona pandemic were observed by the participants, we used the 32-item Perception of Change Questionnaire (PCQ), with has good psychometric properties (Cronbach's alpha = 0.91) (4). The instrument differentiates five main factors: (1) Nature/Silence/Contemplation (seven items, Cronbach's alpha = 0.87); (2) Spirituality (five items, Cronbach's alpha = 0.83); (3) Relationships (six items, Cronbach's alpha = 0.80); (4) Reflection on life (three items, Cronbach's alpha = 0.74); (5) Digital media usage (three items, Cronbach's alpha = 0.74), and an additional three-item factor termed Restrictions (Cronbach's alpha = 0.78) (4). The internal consistency of the respective factors in this sample is partially better than in the validation sample (Cronbach's alphas = 0.88, 0.86, 0.84, 0.74, 0.77, and 0.83).

The items were introduced by the phrase “Due to the current situation…,” which referred to the COVID-19 pandemic. Representative items are “I pay more attention to what's really important in life,” “I perceive the relationship with my partner/family more intensely,” “I perceive nature more intensely,” “I am more concerned about the meaning and purpose of my life,” and “I have confidence in a higher power that supports me.” Agreement or disagreement was scored on a five-point scale (0—does not apply at all; 1—does not truly apply; 2—neither yes or no; 3—applies quite a bit; 4—applies very much).

#### Well-Being

To assess participants' well-being, we used the WHO-Five well-being Index (WHO-5) (23). Representative items are “I have felt cheerful and in good spirits” or “My daily life has been filled with things that interest me.” Respondents assess how often they had the respective feelings within the last 2 weeks, ranging from “at no time” (0) to “all of the times” (5). Here we report the sum scores ranging from 0 to 25. Scores <13 would indicate reduced well-being or even depressive states. In comparison, the alternative WHO-5 sum scores referred to a 100% level [0–100], which is also used in literature, scores <50 are indicative for reduced well-being, while scores < 28 are indicative for clinical depression (24). The internal consistency of this well-established instrument in this sample is very good (Cronbach's alpha = 0.91).

#### Life Satisfaction

Life satisfaction was measured using the Brief Multidimensional Life Satisfaction Scale (BMLSS) (25). The items of the BMLSS address intrinsic (oneself, life in general), social (friendships, family life), external (work situation, where one lives), and prospective dimensions (financial situation, future prospects) of life satisfaction as a multifaceted construct. All items were introduced by the phrase “I would describe my satisfaction with … as …. ”. Scoring ranges from very dissatisfied (0) to very satisfied (6). The internal consistency of the instrument was found to be good in the validation study (Cronbach's alpha = 0.87). In this study, the 10-item version was employed that includes satisfaction with the health situation and abilities to deal with daily life concerns (BMLSS-10). The instruments' internal consistency is good in this sample, too (Cronbach's alpha = 0.86). We further addressed participants' satisfaction with support by religious community with the same scoring.

#### Perception of Burden

Perceived restrictions of daily life, of being under pressure/stressed, anxiety/insecurity, loneliness/social isolation, and restrictions of financial–economic situation due to the corona pandemic were measured with five numeric rating scales (NRS), ranging from 0 (not at all) to 100 (very strong) as described (4). These five variables can be combined to a factor termed “Stressors” (5NRS) with good internal consistency (Cronbach's alpha = 0.80). The instruments' internal consistency in this sample is good, too (Cronbach's alpha = 0.82).

#### Indicators of Spirituality

Perceptions of wondering awe and subsequent gratitude is a perceptive aspect of spirituality which is also relevant to less or non-religious persons (26). To address times of pausing for astonishment or “wonder” in specific stations (mainly in the nature), we measured perceived awe and subsequent feelings of gratitude as a perceptive aspect of spirituality with the seven-item Awe/Gratitude scale (GrAw-7) (26). This scale has good psychometric properties (Cronbach's alpha = 0.82) and uses items such as “I stop and then think of so many things for which I'm really grateful,” “I stop and am captivated by the beauty of nature,” “I pause and stay spellbound at the moment,” and “In certain places, I become very quiet and devout.” Thus, Awe/Gratitude operationalized in this way is a matter of an emotional reaction toward an immediate and “captive” experience. All items were scored on a four-point scale (0—never; 1—seldom; 2—often; 3—regularly) and finally transferred to a 100-point scale. The instruments' internal consistency in this sample is good (Cronbach's alpha = 0.86).

To measure also more specific forms of religiosity, we added item A37 from the Reliance on God's Help scale (27), which asks whether faith is a strong hold in difficult times. Agreement or disagreement was scored on a three-point scale (0—disagreement; 1—indifference; 2—agreement). This item was used as a differentiating variable to assess intrinsic religiosity in terms of an attitude.

The frequency of spiritual/religious practices such as meditating or praying was assessed with a 4-grade scale ranging from never, to at least once per month, at least once per week, and at least once per day as described (4).

#### Corona Pandemic Irritations

Several persons reported that they were “Irritated or unsettled by different statements about the danger and the course of the corona infection in the public media” (1, 3). Agreement to this statement was scored from not at all, a little, somewhat, to very much.

#### Frequency of Physical Activity

Health behaviors such as physical activity/sporting and walking outside in the nature were measured with a four-grade scale (never, at least once per month, at least once per week, at least once per day) as described (4).

### Statistical Analysis

Descriptive statistics for demographic variables and for factors are presented as frequencies for categorical variables and as mean (± standard deviation, SD) for numerical variables. Between-group comparisons for categorical variables were performed with Pearson's Chi^2^ Independence Test and for numerical variables with the non-parametric Mann–Whitney-*U* hypothesis test. Analyses of variance (ANOVA) and linear regression analyses with stepwise variable selection based on probabilities were computed with SPSS 23.0. Given the exploratory character of this study, we set a stricter significance level at *p* < 0.001 (28).

A post hoc power analysis (G^*^Power 3.1.9.7) was performed to evaluate the significance and power of the statistical analysis. Given the final sample size, the three time measurement periods (three cohorts) considering the alpha level at 0.05, and an overall medium effect size f = 0.25, we were able to achieve a post hoc power of 1.00. In consequence, we can conclude that this data set is appropriate to perform such evaluations. Therefore, we are comfortable to draw the conclusions based on the data analysis.

There were some missing values in the variables with a maximum frequency of 2.6%. Since the percentage of missing data is low, multivariate imputation was applied using the Expectation Maximization (EM) method (29).

With respect to classifying the strength of the observed correlations, we adjusted the recommended thresholds (30) to r > 0.5 as a strong correlation, an r between 0.3 and 0.5 as a moderate correlation, an r between 0.2 and 0.3 as a weak correlation, and r < 0.2 as negligible or no correlation. Hedge's g effect sizes (31) and its confidence intervals (CI) were calculated with software R 4.0.3 package “effsize.” Regarding Hedges' g, we used the following thresholds g > 0.80 large effect, g between 0.50 and 0.80 as medium effect, g between 0.20 and 0.50 as small effect, and g < 0.20 as negligible.

## Results

### Description of the Cohorts

In this study, we enrolled participants from three time periods related to the COVID-19 pandemic: (1) persons directly after the first lockdown (June 2020; *n* = 1,333), (2) persons from the “delighted” summer months July to September 2020 (*n* = 823), and (3) persons at the start of the second wave of the pandemic (October to January, *n* = 625), which includes the second lockdown months December 2020 and January 2021.

Participants from these three cohorts did not significantly differ with respect to gender or family status, but they were younger (~12% younger) in the 3rd cohort (Table 1). Moreover, in this 3rd cohort the proportion of persons without a religious affiliation was significantly higher (~doubled), and the proportion of persons who rely on their faith as a “strong hold in difficult times” was lower (~44% less), and the proportion of those who stated that they have lost their faith because of the pandemic was increasing, too (~5 times higher). Also, the satisfaction with the support by their local religious community (which was moderate) decreased significantly (Table 1).

**Table 1:** Description of the samples (*N* = 2,781).

	**Participants Cohort 1 (June)**	**Participants Cohort 2 (July to September)**	**Participants Cohort 3 (October to January)**	**All participants**	**p-values (Pearson's Chi^**2**^) (Mann–Whitney-U test)**
Proceeding participants (*n*)	1,333	823	625	2,781	
Mean age (years)	48.9 ± 14.1	47.4 ± 13.0	42.7 ± 14.4	47.1 ± 14.1	<0.0001
Gender (%)					n.s.
Women	66.4	69.9	66.4	67.4	
Men	33.6	30.1	33.6	32.6	
Family status (%)					n.s.
Living alone	22.3	21.3	18.9	21.2	
Other forms	77.7	78.7	81.1	78.8	
Religious affiliation (%)					<0.0001
With	78.8	78.0	59.7	74.3	
Without	21.1	22.0	40.3	25.7	
Faith as a strong hold (%)					<0.0001
No	27.0	27.2	53.5	33.1	
Indifferent	31.3	30.3	23.1	29.1	
Yes	41.8	42.5	23.4	37.8	
Satisfaction with support by religious community (0–6)	3.05 ± 1.22	2.98 ± 1.27	2.68 ± 1.44	2.96 ± 1.29	<0.0001
Lost my faith (%)	2.8	3.7	14.7	5.4	<0.0001
COVID-19 testing (%)					<0.0001
Positively tested	0.7	1.0	4.0	1.5	
Negatively tested	11.2	19.9	28.5	17.4	
Not yet tested	88.3	79.8	67.5	81.1	
Irritated by statements about danger and course of the infection in public media (%)	16.9	16.0	18.4	17.0	n.s.
Not at all	37.2	34.1	21.3	32.7	<0.0001
A little	32.3	36.2	25.9	32.0	<0.0001
Somewhat very much	14.3	14.0	35.2	18.9	<0.0001
Well-being (%)					<0.0001
Low (WHO-5 scores < 13)	31.0	30.1	63.5	38.0	
Moderate (WHO-5 scores 13–18)	39.3	38.9	21.9	35.3	
High (WHO-5 scores > 18)	29.7	31.0	14.6	26.7	
Social isolation/loneliness (%)					<0.0001
No loneliness (scores < 10)	33.3	37.7	19.5	31.5	
Somewhat lonely (scores 10–50)	51.4	48.5	34.2	46.7	
Stronger loneliness (scores > 50)	15.3	13.9	46.2	21.8	
Health behavior (0–3)					
Frequency sporting activities	1.83 ± 0.90	1.74 ± 0.91	1.45 ± 1.05	1.72 ± 0.95	<0.0001
Frequency walks in nature	2.09 ± 0.74	2.03 ± 0.76	1.94 ± 0.92	2.04 ± 0.79	0.002
Frequency meditation	1.20 ± 1.19	1.02 ± 1.16	0.63 ± 1.01	1.02 ± 1.16	<0.0001
Frequency praying	1.59 ± 1.29	1.49 ± 1.27	0.82 ± 1.19	1.40 ± 1.30	<0.0001

Parallel to the number of infected persons in Germany which increased from October 2020 (31.5/100,000) to January 2021 (151/100,000), there was an increase of persons who are still “irritated by statements about danger and course of the infection in public media,” who feel lonely and socially isolated, and who have lower well-being (Table 1).

The frequency of participants' sporting activities started to decline in cohort 2 [Hedges' g = 0.10 (0.01–0.19)] and was lowest in cohort 3 [Hedges' g = 0.40 (0.30–0.50)] (Table 1). The frequency of walking in nature was similar in cohorts 1 and 2 [Hedges' g = 0.07 (−0.01–0.16)] and declined in cohort 3 [Hedges' g = 0.17 (0.07–0.28)]. The frequency of meditation practices started to decline in cohort 2 [Hedges' g = 0.15 (0.06–0.24)] with a similar trend of praying [Hedges' g = 0.08 (−0.01–0.17)], while both spiritual practices were lowest in cohort 3 [Hedges' g = 0.50 (0.40–0.60) and g = 0.61 (0.51–0.72), respectively]. Thus, the decreases were moderate for meditating and praying and less than small for walking and a bit more only for sporting activities.

### Perceived Changes in the Cohorts

There is constant decline of perceived positive changes, starting in the summer months, which was lowest in cohort 3, while perceived Restrictions were increasing in cohort 3 (Table 2). In the participants of the 3rd cohort, the scores of Nature/Silence/Contemplation and Digital media usage indicated that they were not perceiving the respective attitudes and behaviors anymore, while positive changes in terms of Spirituality were not generally perceived in cohort 1's participants and scored much lower now in cohort 3. Although positive changes of Relationships were declining, these are still positively perceived in cohort 3 (Table 2).

**Table 2:** Perceived changes in persons from cohorts 1–3.

		**Perceived changes (PCQ)**					
		**Nature/Silence/Contemplation**	**Spirituality**	**Relationships**	**Reflection on life**	**Digital media usage**	**Restrictions**
All participants	Mean	54.56	38.80	62.01	52.23	53.40	47.61
	SD	21.25	25.92	19.18	24.88	23.74	29.17
Cohort 1	Mean	58.49	43.56	64.99	52.48	56.07	42.70
	SD	20.07	25.25	17.99	24.73	22.85	26.54
Cohort 2	Mean	55.68	39.43	62.72	51.00	52.51	41.42
	SD	20.51	25.07	18.94	24.70	23.99	27.27
Cohort 3	Mean	44.70	27.83	54.71	53.32	48.87	66.24
	SD	21.60	25.19	20.06	25.41	24.54	29.30
F- value		97.5	83.4	64.7	1.7	20.7	186.9
*p*-value		<0.0001	<0.0001	<0.0001	n.s.	<0.0001	<0.0001
Hedges' d (CI) Cohort 1 vs. 3		0.67 (0.57 to 0.77)	0.62 (0.52 to 0.72)	55 (0.45 to 0.65)	−0.03 (−0.13 to 0.06)	0.31 (0.21 to 0.40)	−0.86 (−0.96 to −0.76)

Because the proportion of persons lacking a religious affiliation was significantly higher in cohort 3, which cannot solely be attributed to the number of persons who have lost their faith because of the pandemic, we analyzed whether or not religious and non-religious participants differ in their perceptions and behaviors within time. As shown in Table 3, non-religious persons of cohort 1 were scoring significantly lower for Spirituality than religious persons, and weakly lower also for Relationships, and for Reflection of life, but not for Nature/Silence/Contemplation, Digital media usage, or Restrictions. In cohort 2, perceived changes in Relationships scored much lower in non-religious compared to religious participants, and thus the difference in their scores is statistically significant. Interestingly, Spirituality scores decreased, too, in religious persons of cohort 2 and thus the differences between both groups are less pronounced than in cohort 1. In cohort 3, there is a decline of most perceptions in both groups, stronger though in non-religious persons (Table 3): perceived changes in Nature/Silence/Contemplation had become significantly different, while the previous differences for Spirituality and Relationships remained statistically significant. Thus, while the differences for Spirituality are not surprising, there are further differences within the course of time related to Relationships in cohorts 2 and 3, and in cohort 3 also for Nature/Silence/Contemplation which can be attributed to stronger declines in non-religious persons.

**Table 3:** Perceived changes in religious and non-religious persons differentiated within the three cohorts.

		**Nature/Silence/Contemplation**	**Spirituality**	**Relationships**	**Reflection on life**	**Digital media usage**	**Restrictions**
**All cohort 1** (*n =* 1,333)	Mean	58.49	43.56	64.99	52.48	56.07	42.70
	SD	20.07	25.25	17.99	24.73	22.85	26.54
Religious (*N =* 1,050, 79%)	Mean	58.80	47.03	65.63	53.33	56.57	42.90
	SD	19.65	24.03	17.48	24.18	23.00	26.25
Non-religious (*n =* 283, 21%)	Mean	57.33	30.69	62.57	49.35	54.21	41.96
	SD	21.56	25.53	19.59	26.48	22.25	27.62
F-value		1.20	100.4	6.49	5.78	2.28	0.28
*p*-value		n.s.	<0.0001	0.011	0.016	n.s.	n.s.
Hedges' g (CI)		0.07 (−0.06 to 0.20)	0.67 (0.54 to 0.80)	0.17 (0.04 to 0.30)	0.16 (0.03 to 0.29)	0.10 (−0.03 to 0.23)	0.03 (−0.10 to 0.17)
**All cohort 2** (*n =* 823)	Mean	55.68	39.43	62.72	51.00	52.51	41.42
	SD	20.51	25.07	18.94	24.70	23.99	27.27
Religious (*N =* 642, 78%)	Mean	56.41	42.48	63.72	51.64	52.15	40.36
	SD	19.86	24.47	18.47	24.26	23.73	26.70
Non-religious (*n =* 181, 22%)	Mean	53.08	28.59	59.19	48.76	53.78	45.21
	SD	22.53	24.23	20.16	26.17	24.90	28.96
F-value		3.73	45.71	8.17	1.92	0.64	4.50
*p-*value		n.s.	<0.0001	0.004	n.s.	n.s.	0.034
Hedges' g (CI)		0.16 (−0.01 to 0.33)	0.57 (0.40 to 0.73)	0.24 (0.07 to 0.40)	0.12 (−0.05 to 0.28)	−0.07 (−0.23 to 0.10)	−0.18 (−0.34 to 0.01)
**All cohort 3** (*n =* 625)	Mean	44.70	27.83	54.71	53.32	48.87	66.24
	SD	21.60	25.19	20.06	25.41	24.54	29.30
Religious (*N =* 373, 60%)	Mean	47.42	33.56	56.61	55.40	49.65	63.77
	SD	21.17	26.02	19.62	25.19	25.22	30.58
Non-religious (*n =* 252, 40%)	Mean	40.69	19.34	51.89	50.26	47.71	69.91
	SD	21.64	21.27	20.41	25.47	23.50	26.94
F-value		14.93	51.90	8.43	6.21	0.90	6.67
p-value		<0.0001	<0.000	0.004	0.013	n.s.	0.010
Hedges' g (CI)		0.31 (0.15 to 0.47)	0.57 (0.42 to 0.75)	0.23 (0.07 to 0.40)	0.20 (0.04 to 0.36)	0.08 (−0.08 to 0.24)	−0.21 (−0.37 to −0.05)

We also asked whether participants fear for the future (additional item c28) and/or to have “hope that we (“afterwards”) as global mankind will pay more attention to each other” (additional item c25). Fear for future increased significantly (*p* < 0.0001, Chi^2^) from 29% (cohort 1) to 36% (cohort 2) and to 68% (cohort 3). In line with this increase of fear, participants' hope for a more conscious global mankind decreased from 55 to 47% and finally to 28% in cohort 3; this decline is statistically significant (*p* < 0.001, Chi^2^). However, in the whole sample the motivation to start working to ensure that “the world becomes fairer in the future” (additional item c26) was stable in cohorts 1 and 2 (64% to 63%) and significantly (*p* < 0.0001, Chi^2^) decreasing in cohort 3 (48%).

### Indicators of Quality of Life in the Three Cohorts

Along with the increase of infected persons from October 2020 to January 2021, there was a decrease of well-being, life satisfaction, and Awe/Gratitude in cohort 3 participants, while these variables had been quite stable in cohorts 1 and 2 (Table 4). Similarly, while the stressor scores were similar in cohorts 1 and 2, the stressors increased in cohort 3 participants. Detailed analyses revealed that the strongest increases were due to the perception of being restricted in daily life (which scored highest), of being under pressure, and the feeling of being lonely/socially isolated, while burdening financial situation (which scored lowest), and feelings of being under pressure/anxious were increasing less strongly (Table 4).

**Table 4:** Stressors and indicators of quality of life in persons from cohorts 1 to 3.

		**Restricted in daily life (NRS1)**	**Under pressure/stressed (NRS2)**	**Anxious/Insecure (NRS3)**	**Lonely/Socially isolated (NRS4)**	**Burdening financial situation (NRS5)**	**Stressors (5NRS)**	**Well-being (WHO-5)**	**Life satisfaction (BMLSS-10)**	**Awe/Gratitude (GrAw-7)**
All participants	Mean	52.30	41.68	26.73	29.53	21.43	34.35	13.88	65.32	61.74
	SD	28.76	31.98	27.23	31.38	30.57	22.93	5.96	17.43	19.65
Cohort 1	Mean	47.50	35.52	23.39	24.51	17.88	29.76	14.96	67.93	64.89
	SD	25.96	29.29	24.01	27.35	27.50	20.00	5.26	15.58	17.98
Cohort 2	Mean	46.49	37.76	25.02	22.59	16.04	29.58	14.95	67.73	63.03
	SD	26.56	29.30	25.29	26.52	26.39	19.46	5.29	16.16	18.28
Cohort 3	Mean	70.22	60.19	36.16	49.56	36.13	50.43	10.16	56.55	53.31
	SD	30.04	33.98	33.39	36.74	36.78	25.60	6.65	19.79	22.28
F- value		176.1	148.6	50.7	185.1	100.5	231.0	176.9	110.0	80.8
*p*-value		<0.0001	<0.0001	<0.0001	<0.0001	<0.0001	<0.0001	<0.0001	<0.0001	<0.0001
Hedges' d (CI) cohort 1 vs. 3		−0.83 (−0.93 to −0.73)	−0.80 (−0.90 to −0.70)	−0.47 (−0.56 to −0.37)	−0.82 (−0.92 to −0.72)	−0.60 (−0.69 to −0.50)	−0.94 (−1.04 to −0.84)	0.83 (0.74 to 0.93)	0.67 (0.57 to 0.76)	0.60 (0.50 to 0.70)

Again, we compartmentalized whether or not non-religious and religious participants from cohorts 1 to 3 differed with respect to their quality-of-life indicators. As shown in Table 5, at the end of the first lockdown (cohort 1), religious and non-religious persons' scores of quality-of-life indicators were not that much different. During the summer months (cohort 2), non-religious participants' life satisfaction and Awe/Gratitude scores started to decline, while these variables were similar compared to cohort 1 data in religious persons. Then in cohort 3, perceptions of both religious and non-religious persons changed similarly: particularly their life satisfaction decreased, and thus their scores did not differ significantly anymore. In addition, during the second wave of the pandemic, Awe/Gratitude decreased also in religious participants, although the difference between religious and non-religious participants remains statistically significant. However, well-being and life satisfaction of cohort 3 participants with or without a religious affiliation did not differ significantly and weakly only for the stressor scores.

**Table 5:** Quality-of-life indicators in religious and non-religious persons differentiated within the three cohorts.

		**Stressors (5NRS)**	**Well-being (WHO-5)**	**Life satisfaction (BMLSS-10)**	**Awe/Gratitude (GrAw-7)**
**All cohort 1** (*n =* 1,333)	Mean	29.76	14.96	67.93	64.89
	SD	20.00	5.26	15.58	17.98
Religious (*N =* 1,050, 79%)	Mean	29.17	14.96	68.29	65.05
	SD	19.21	5.23	14.98	17.97
Non-religious (*n =* 283, 21%)	Mean	31.96	14.92	66.59	64.30
	SD	22.58	5.40	17.59	18.03
F-value		4.37	0.01	2.65	0.39
*p*-value		0.037	n.s.	n.s.	n.s.
Hedges' g (CI)		−0.14 (−0.27 to −0.01)	0.01 (−0.12 to 0.14)	0.11 (−0.02 to 0.24)	0.04 (−0.09 to 0.17)
**All cohort 2** (*n =* 823)	Mean	29.58	14.95	67.73	63.03
	SD	19.46	5.29	16.16	18.28
Religious (*N =* 642, 78%)	Mean	29.19	15.16	68.53	64.04
	SD	18.90	5.03	15.67	18.23
Non-religious (*n =* 181, 22%)	Mean	30.96	14.22	64.91	59.42
	SD	21.31	6.10	17.53	18.06
F-value		1.17	4.42	7.14	9.10
*p*-value		n.s.	0.036	0.008	0.003
Hedges' g (CI)		−0.09 (−0.26 to 0.07)	0.18 (0.01 to 0.34)	0.22 (0.06 to 0.39)	0.25 (0.09 to 0.42)
**All cohort 3** (*n =* 625)	Mean	50.43	10.16	56.55	53.31
	SD	25.60	6.65	19.79	22.28
Religious (*N =* 373, 60%)	Mean	48.54	10.55	57.71	55.75
	SD	25.46	6.62	19.60	21.71
Non-religious (*n =* 252, 40%)	Mean	53.22	9.58	54.83	49.70
	SD	25.59	6.66	19.98	22.66
F-value		5.05	3.20	3.30	11.30
*p*-value		0.025	n.s.	n.s.	0.001
Hedges' g (CI)		−0.18 (−0.34 to −0.02)	0.14 (−0.01 to 0.30)	0.14 (−0.01 to 0.30)	0.27 (0.11 to 0.43)

To further clarify the impact of being non-religious on these variables, we also performed regression analyses. As shown in Table 6, both stressors and life satisfaction were predicted best by well-being, with only some marginal effects of age, gender, and being non-religious. The best predictor of Awe/Gratitude was well-being and gender, followed by age, and a further weak influence of being non-religious. Thus, being non-religious may have some small influence on a person's quality-of-life indicators but is not what mostly adds to the negative shifts in persons quality-of-life indicators.

**Table 6:** Regression analyses with constant influencing variables age, gender, lack of religious affiliation, and well-being.

**Dependent variables**	**Influencing variables**	**Beta**	**T**	***p***
Stressors (5NRS) R^2^ = 0.43 F = 518.2, *p* < 0.0001	(Constant)		46.436	<0.0001
	Age	−0.068	−4.547	<0.0001
	Gender	−0.038	−2.644	0.008
	No religious affiliation	0.055	3.820	<0.0001
	Well–being	−0.625	−41.806	<0.0001
Life satisfaction (BMLSS-10) R^2^ = 0.42 F = 483.9, *p* < 0.0001	(constant)		32.996	<0.0001
	Age	−0.020	−1.322	0.186
	Gender	0.005	0.372	0.710
	No religious affiliation	−0.049	−3.325	0.001
	Well–being	0.641	42.297	<0.0001
Awe/Gratitude (GrAw-7) R^2^ = 0.20 F = 168.6, *p* < 0.0001	(Constant)		28.323	<0.0001
	Age	0.176	9.925	<0.0001
	Gender	−0.213	−12.335	<0.0001
	No religious affiliation	−0.079	−4.600	<0.0001
	Well-being	0.321	18.074	<0.0001

### Predictors of Perceived Changes

What are the predictors of the perceived changes (as dependent variables) due to the pandemic? To answer this question, we performed linear regression analyses with a stepwise selection method based on probabilities with the following influencing variables: age groups, well-being (WHO-5), life satisfaction (BMLSS-10), Awe/Gratitude (GrAw-7), Stressors (5NRS), loneliness/social isolation (NRS4), faith as a strong hold, meditation, and praying—and included the cohort as an influencing variable, too.

As shown in Table 7, the best predictors of Nature/Silence/Contemplation as dependent variable were Awe/Gratitude and meditation (which explained 24% of variance), followed by well-being (which added further 4%), and then cohort and Faith as a strong hold (which added 0.6% only, and are thus irrelevant).

**Table 7:** Predictors of perceived changes (stepwise regression analyses).

		**Beta**	**T**	***p***
Dependent variable: Nature/Silence/Contemplation Model 5: F = 197.2, *p* < 0.0001; R^2^ = 0.29				
	(Constant)		14.681	<0.0001
	Awe/Gratitude (GrAw-7)	0.225	10.840	<0.0001
	Meditation	0.209	10.607	<0.0001
	Well-being (WHO-5)	0.188	9.938	<0.0001
	Cohort	−0.089	−4.903	<0.0001
	Faith as a stronghold	0.082	4.235	<0.0001
Dependent variable: Spirituality Model 8: F = 439.2, *p* < 0.0001; R^2^ = 0.59				
	(Constant)		2.742	0.006
	Praying	0.328	17.302	<0.0001
	Faith as a stronghold	0.315	16.739	<0.0001
	Meditation	0.196	12.879	<0.0001
	Awe/Gratitude (GrAw-7)	0.110	6.855	<0.0001
	Loneliness/Social Isolation (NRS4)	0.058	3.933	<0.0001
	Cohort	−0.064	−4.681	<0.0001
	Live satisfaction (BMLSS-10)	−0.048	−3.087	0.002
	Age groups	0.035	2.479	0.013
Dependent variable: Relationships Model 5: F = 100.2, *p* < 0.0001; R^2^ = 0.17				
	(Constant)		13.556	<0.0001
	Awe/Gratitude (GrAw-7)	0.266	12.521	<0.0001
	Life satisfaction (BMLSS-10)	0.170	7.225	<0.0001
	Cohort	−0.112	−5.651	<0.0001
	Faith as a stronghold	0.077	3.809	<0.0001
	Stressors (5NRS)	0.076	3.301	0.001
Dependent variable: Reflections of life Model 9: F = 71.0, *p* < 0.0001; R^2^ = 0.21				
	(Constant)		8.223	<0.0001
	Meditation	0.115	5.428	<0.0001
	Loneliness/Social Isolation (NRS4)	0.168	6.374	<0.0001
	Awe/Gratitude (GrAw-7)	0.241	10.686	<0.0001
	Life satisfaction (BMLSS-10)	−0.136	−5.446	<0.0001
	Praying	0.055	2.073	0.038
	Well-being (WHO-5)	−0.086	−3.168	0.002
	Age groups	0.077	3.906	<0.0001
	Faith as a stronghold	0.073	2.768	0.006
	Stressors (5NRS)	0.071	2.335	0.020
Dependent variable: Digital media usage Model 6: F = 27.4, *p* < 0.0001; R^2^ = 0.06				
	(Constant)		11.233	<0.0001
	Mediation	0.140	6.340	<0.0001
	Awe/Gratitude (GrAw-7)	0.092	3.913	<0.0001
	Loneliness/Social Isolation (NRS4)	0.076	2.649	0.008
	Cohort	−0.099	−4.664	<0.0001
	Life satisfaction (BMLSS-10)	0.072	2.868	0.004
	Stressors (5NRS)	0.074	2.344	0.019
Dependent variable: Perceived Restrictions Model 7: F = 383.5, *p* < 0.0001; R^2^ = 0.53				
	(Constant)		9.074	<0.0001
	Stressors (5NRS)	0.321	13.581	<0.0001
	Loneliness/Social Isolation (NRS4)	0.304	14.929	<0.0001
	Well-being (WHO-5)	−0.100	−4.816	<0.0001
	Cohort	0.079	5.227	<0.0001
	Life satisfaction (BMLSS-10)	−0.083	−4.405	<0.0001
	Meditation	−0.043	−2.961	0.003
	Age group	0.034	2.312	0.021

*Variables included in the stepwise regression analyses: age groups, Well-being (WHO-5), life satisfaction (BMLSS-10), Awe/Gratitude (GrAw-7), Stressors (5NRS), Loneliness/Social isolation (NRS4), Faith as a stronghold, mediation, praying, cohort*.

The best predictors of Spirituality were praying and Faith as a strong hold (which together explained 53% of variance), followed by frequency of meditation and Awe/Gratitude (which add 6% of explained variance), and four further variables, among them cohort (which together added further 0.6% of explained variance and are thus irrelevant).

The Relationships factor was predicted best by Awe/Gratitude and life satisfaction (which explain 12% of variance), followed by cohort, Faith as a strong hold, and Stressors (which together added further 2%). However, this predictor model is quite weak (*R*^2^ = 0.17) and findings may be seen as a hint only.

Reflection of life was predicted best by meditation, loneliness/social isolation, and Awe/Gratitude (which explained together 15% of variance), followed by six further variables, including being non-religious (which together explained further 5% of variance).

The predictor model of Digital media usage was much too weak (*R*^2^ = 0.06) to rely on.

The best predictors of perceived Restrictions were Stressors and loneliness/social isolation (which explain 50% of variance), followed by four further variables, including cohort (which added 3% of explained variance).

## Discussion

Directly after the first lockdown which took about 3 months, people noticed also positive changes in their attitudes and behaviors. They were more aware of their relationships and intensified and valued them more than before, and they were more often outdoors for some walks, perceived nature more intensively, and consciously took more time for silence, enjoyed quiet times of reflection, were more attentive to what they deemed is really important in life, and used these extra times to reflect on meaning and purpose in life (4). This mindful approach to challenging life situations was shown to have a protective effect on health behaviors (32). To overcome social isolation, digital media usage was intensified to connect with friends and to participate in the world via internet offers (4), while interest in spiritual issues was not intensified, apart from participants' high confidence in a “higher power” (God) that supports them. Further, participants stated they have hope that (“afterwards”) we as global mankind will pay more attention to each other and stick together, and that they intend to start working to ensure that the world becomes fairer in the future (4). Awe/Gratitude, which addresses the ability to stop in wondering awe in specific situations with subsequent feelings of gratitude (26, 33), was the best predictor for most of these changes. This is a perceptive aspect of spirituality which does not necessarily require a religious denomination but is likewise experienced by people who would rather identify themselves as non-religious (33). Nevertheless, it indicates that a person's spirituality may influence their specific perceptions also in difficult times of a pandemic. Further, particularly the frequency of meditation practices (and praying) was related to Nature/Silence/Contemplation, indicating that contemplative/reflexive practices may sensitize for the awareness of specific perceptions (4). As these positively perceived changes were only to some extent related to well-being (weakly only to Nature/Silence/Contemplation), they might “represent an independent quality of relevance in their life” (4). A key issue will thus be whether the observed positive changes of attitudes and behaviors are short-term effects only or may help to cope with the further course of the pandemic in the long run, too.

**Figure 1:**
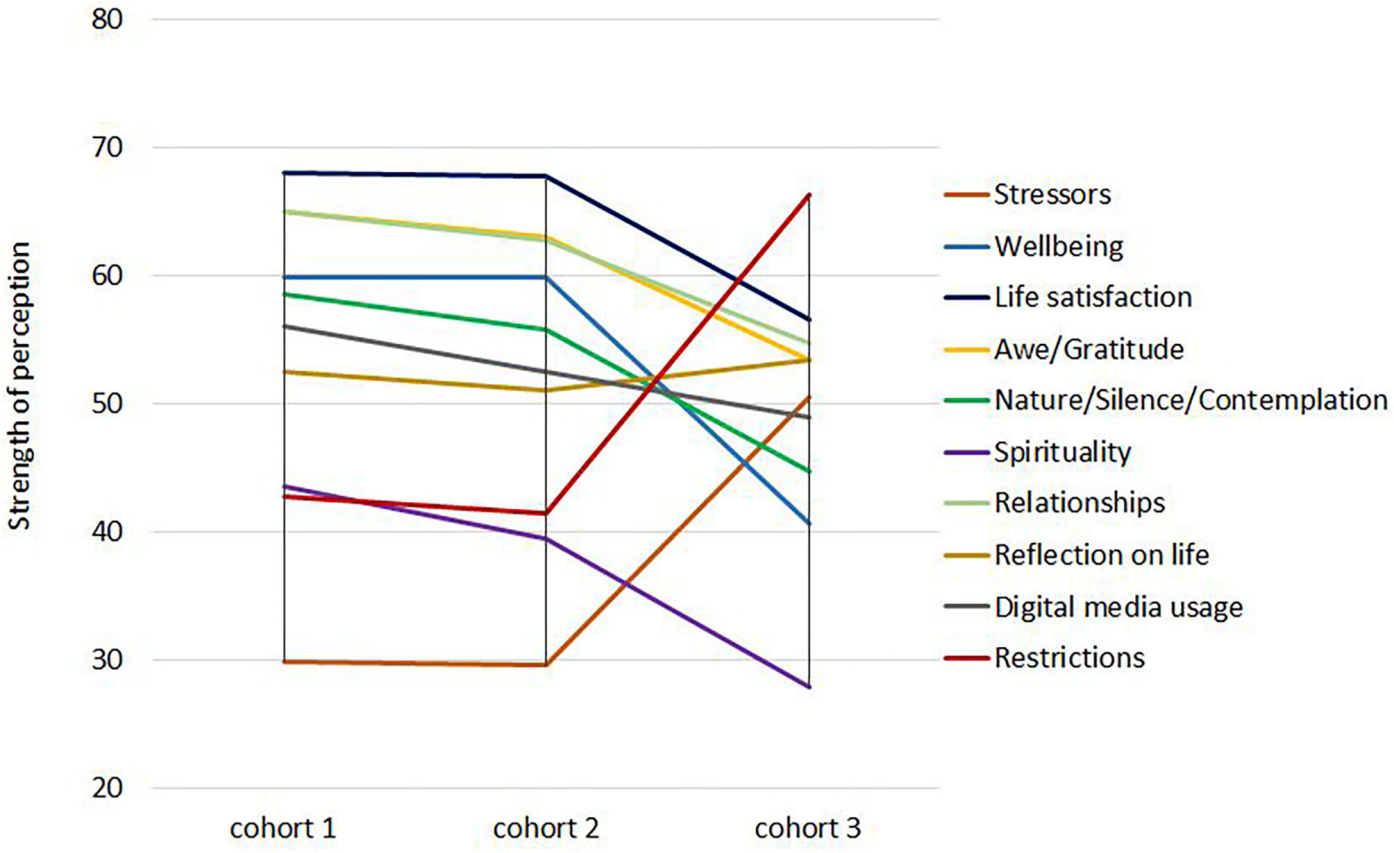
Strength of perceptions in the three cohorts.

Findings from the three cohorts analyzed herein indicate that several perceptions, attitudes, and behaviors have changed, particularly during the second wave of the pandemic: Participants' well-being and life satisfaction decreased and perceived burden (“stressors”) increased; most perceived positive perceptions as well as health behaviors (sporting activities and walks in nature) and hope for positive changes have declined (Figure 1). These changes are embedded in the phenomena and consequences of societal disruption due to the public COVID-19 measures, as described analogously for the American society, including the high numbers of deaths, while expecting the second wave of autumn 2020: “The necessary social distancing and quarantine measures implemented as mitigation strategies have significantly amplified emotional turmoil by substantially changing the social fabric by which individuals, families, communities, and nations cope with tragedy. The effect is multidimensional disruption of employment, finances, education, health care, food security, transportation, recreation, cultural and religious practices, and the ability of personal support networks and communities to come together and grieve.” (8). All of these elements will have their diffuse share in the perceived changes and in the decrease of participants' personal spiritual or religious practices.

Even though it may be true that walking in nature or sporting activities during late autumn and winter times (at the start of the second wave and its second lockdown) are not as attractive as in spring or summer time (directly after the first lockdown), and thus the frequency of engagement is slightly decreasing, it does not explain why also meditation practices and praying decreased in cohort 3, because these can be performed indoors, too, of course. A reason could be the larger fraction of non-religious persons in cohort 3, or a weakening of motivation and trust. Also within the group of religious persons, the frequency of meditation [Hedges' g = 0.50 (0.40–0.60)] and praying [Hedges' g = 0.61 (0.51–0.72)] was decreasing from cohorts 1 to 3, yet stronger in non-religious participants [Hedges' g = 0.73 (0.54–0.91) and g = 0.43 (0.25–0.61), respectively]. This indicates, first, that there is a common strong impact of the second wave of the pandemic (including the second lock down) on these behaviors, which affect both religious and non-religious persons. Also, religious persons may have lost some of their motivation or confidence to rely on their spirituality as a resource and were less engaged to meditate or pray. In line with this, even within the religious participants, agreement that they rely on their faith as a strong hold decreased from 46 to 32% and from 26 to 11% in nominally non-religious persons. A second indication hints to the use of meditation which dropped strongly among non-religious participants between the first and third cohorts (g = 0.73), while prayer dropped less (g = 0.43); in comparison, the decrease of meditation among religious persons was moderate (g = 0.50), of prayer however stronger (g = 0.61). This may indicate that in the first period, recommendations or stimuli for times to reflect (or even times for meditation) were received positively by non-religious persons; the longer the pandemic has taken, however, the stronger many of them may have got tired of it. The religious cohorts show a similar effect for praying which they may have intensified during the first lockdown which they neither kept up nor started again during the second lockdown. This is a global observation which would call for a more qualitative investigation of which personal meanings both groups across the cohorts tend to associate with meditation and praying personally. Further, the quite low satisfaction with the support of their local religious communities during the pandemic was significantly declining in the second wave of the pandemic, too. It seems that the expectations that the own faith (in terms of public and private religiosity) can be utilized as a resource to rely on, was shaken in several persons, also in religious people.

In the light of our findings, one has to state that the decline of positive perceptions, particularly for Nature/Silence/Contemplation, Spirituality, and Relationships, might be due to a lack of motivation and courage, too, or a downgrading (adaptation) of emotional engagement as a consequence of the generalized tiring burden of the pandemic measures. Participants' hope that the “easiness” of the summer months will continue was gone, and they were facing the reality of the second wave of the pandemic with all its social restrictions once again. Thus, perceived restrictions and stressors were increasing, and well-being and life satisfaction were declining. The respective dynamics are depicted in Figure 1.

Awe/Gratitude was confirmed as the best predictor of the perceived positive changes related to the perceptions, particularly Nature/Silence/Contemplation, while the cohort itself as an independent variable had only marginal influence on the respective scores. Nevertheless, this ability to stop in wondering awe and gratitude is decreasing during the second wave of the pandemic, too, and can be utilized as a buffering resource only in part.

It was striking that directly after the first lockdown idealistic thoughts were of relevance (these can be seen as an indicator of hope). On the one hand, people had fear for the future but nevertheless had hope that we (“afterwards”) as global mankind will pay more attention to each other. However, during the course of the pandemic the fears were increasing and hope declining. Also, the idealistic motivation to start working to ensure that the world becomes fairer in the future decreased in cohort 3. These are further indicators that the pandemic has impact also on future perspectives, hopes, ideals, and meaning constructs. Persons perceiving in this way are not “sick” and would necessarily require psychological/medical help, but they are nevertheless heavily burdened and require support which is so far not provided. A clear perspective seems to be critical in order to be able to persevere. Rather, all idealistic goals at what time point the pandemic could be “mastered” were not reliable. Setbacks, new virus mutations, and the beginning of the 3rd wave with again increasing death rates give rise to little hope in the general population. Thus, there is a need for new and not yet defined public health communities that could focus on persons which are affected in their physical, mental, social, and spiritual health and well-being due to the pandemic. The psychological and social outcomes of the pandemic experiences are so far not yet clear and require early planning processes. Two important topics to deal with in this context are risk perception and prevention. In a sample from China, Ding et al. (34) have shown that participants' risk perception was associated with depressive states in a differentiated way: *affective* risk perception was positively associated with depression, while *cognitive* risk perception was negatively associated. In addition, “support of prevention and control policies” was inversely related with depression. These findings would imply that health policies should provide reliable information about groups at risk and about general risk protection strategies to reduce fears and worries, and to maintain peoples' health and well-being. Further, government responses to cope with public crises are required to be meaningful and comprehensible in order to get public acceptance, thereby avoiding insecurity and anxiety. Further, a study at the start of the pandemic in Italy revealed that person-related psychological factors may play an important role for risk perceptions and psychological interventions, i.e., “empathy, self-efficacy, and imagination” (35). These resources could help to empower persons in difficult situations. In this study, it was the ability to mindfully stop in wondering awe with subsequent feelings of gratitude that predicted the positive perceptions—a resource that could be trained. However, in several situations peoples' affective reactions have a stronger impact on their behaviors than cognitive approaches, and dealing with this affective reality remains an important task for health care policies.

### Limitations

This is not a longitudinal study with the same participants but with different cohorts at different time-points. Therefore, one has to consider differences in sociodemographic data. These were considered in this evaluation, particularly the higher proportion of non-religious persons in cohort 3. We can underline that being non-religious would account for 1% only of the variance of well-being, life satisfaction, awe/gratitude, Nature/Silence/Contemplation, and 1.5% of the stressor variance.

Further, the study was performed as an online survey with a snowball sampling method and we do not assume that the findings are representative for all groups in German societies as we may not have reached all social groups in a similar manner. To avoid a bias, we have excluded all religious persons living in monastic structures (brothers and sisters, monks, and nuns) which were participating predominantly directly after the first lockdown.

## Conclusions

Findings indicate that several perceptions, attitudes, and behaviors have changed, particularly during the second wave of the pandemic, which had a strong influence on psychological health. Well-being and life satisfaction decreased along with perceived restrictions. The ability to perceive the Sacred in life (in terms of mindful awareness) was confirmed as the best predictor of perceived positive changes, particularly on Nature/Silence/Contemplation. However, this resource may not buffer against the negative outcomes of the pandemic but helps to recognize the still positive aspects in life in terms of an awareness shift to protect own abilities to positively participate in life concerns. Whether this ability could be trained to better cope with the pandemic restrictions remains to be shown. At least it can be stated that spiritual/religious persons may have a benefit in the ability to be more aware for these perceptions than non-spiritual/non-religious persons. However, even this resource was declining in the second wave of the pandemic with its second lockdown. The lack of a positive perspective, that there will be an end of the pandemic, seems to be a highly burdening situation which is difficult to cope with.

## Data Availability Statement

According to the data protection regulations, the data set cannot be made publicly available. Data are however available from the first author upon reasonable request.

## Ethics Statement

Ethical review and approval was not required for this anonymous study on healthy human participants in accordance with the local legislation and institutional requirements. Written informed consent for participation was not required for this study in accordance with the national legislation and the institutional requirements. By filling in the anonymous online questionnaire, interested persons consented to participate.

## Author Contributions

AB designed the study and set up the online survey. TD, JS, and AB initiated the sampling processes. AB and DR undertook statistical analyses. AB, DR, and KB wrote the first draft of the paper. All authors provided feedback and approved the final manuscript.

## Conflict of Interest

The authors declare that the research was conducted in the absence of any commercial or financial relationships that could be construed as a potential conflict of interest.
